# Unraveling the cardiovascular burden of long COVID: symptom profiles, underlying mechanisms, and clinical management insights

**DOI:** 10.3389/fcvm.2026.1786633

**Published:** 2026-06-01

**Authors:** Jie Sheng, Yuchen Song, Aiping Zhang, Minhua Wang, Tao Li, Jianjian Ji

**Affiliations:** 1 Jiangsu Key Laboratory of Pediatric Respiratory Disease, Jiangsu Provincial Research Institute of Chinese Medicine Schools, Nanjing University of Chinese Medicine, Nanjing, China; 2 School of Integrative Medicine, Jiangsu Provincial Research Institute of Chinese Medicine Schools, Nanjing University of Chinese Medicine, Nanjing, China; 3 Clinical lab, Jiangning District Center for Disease Control and Prevention, Nanjing, China; 4 Yixing Hospital of Traditional Chinese Medicine, Yixing, China; 5 Wuxi Affiliated Hospital of Nanjing University of Chinese Medicine, Nanjing University of Chinese Medicine, Wuxi, China

**Keywords:** cardiovascular disease, endothelial dysfunction, epidemiology, long covid, microvascular injury, SARS-CoV-2

## Abstract

**Background:**

Long COVID refers to multisystem symptoms that begin within 3 months of COVID-19 infection and persist for at least 2 months. To this day, Long COVID remains a challenging clinical entity and a substantial global health burden, with cardiovascular sequelae representing a prominent component. Patients frequently report a range of symptoms including chest pain, palpitations, fatigue, and exercise intolerance.

**Objective:**

This mini review aims to synthesize current evidence on the symptom profiles, underlying mechanisms, and clinical management of Long COVID-related cardiovascular complications.

**Methods:**

We conducted a targeted narrative literature search of PubMed/MEDLINE, Web of Science, Scopus, Embase, and Google Scholar for articles published up to January 2026 using combinations of “Long COVID,” “post-acute sequelae of SARS-CoV-2 infection,” “cardiovascular,” “myocarditis,” “endothelial dysfunction,” “microvascular injury,” “dysautonomia,” “vaccination,” and “SARS-CoV-2 variants.” Original studies, systematic reviews, meta-analyses, clinical guidance documents, and selected mechanistic studies were prioritized, whereas non-peer-reviewed preprints and single case reports were included only when they provided unique mechanistic or hypothesis-generating information. Eligibility was based on cardiovascular relevance to Long COVID; studies without post-acute or cardiovascular relevance were excluded.

**Results:**

The evidence indicates that cardiovascular Long COVID is heterogeneous and multifactorial, involving viral persistence, immune dysregulation, endothelial dysfunction, microvascular injury with hypercoagulability, autonomic nervous system dysregulation, and risk modification by acute disease severity, vaccination status, and SARS-CoV-2 variant period. Current management strategies remain primarily symptom-based, with emphasis on cardiovascular risk assessment, mechanism-informed phenotyping, graded rehabilitation, dysautonomia-directed treatment, and multidisciplinary follow-up.

**Conclusions:**

Cardiovascular Long COVID is a heterogeneous burden driven by interacting mechanisms. Current evidence supports subgroup-based risk stratification and mechanism-informed management, while future studies should standardize endpoints and evaluate mechanism-targeted interventions.

## 1 Introduction

The global spread of severe acute respiratory syndrome coronavirus 2 (SARS-CoV-2) initially precipitated a public health emergency dominated by acute respiratory illness and mortality. Nevertheless, as the pandemic progressed, Post-Acute Sequelae of SARS-CoV-2 Infection (PASC), also termed Long COVID, emerged as a major concern, affecting millions worldwide regardless of the severity of the initial infection (1). The World Health Organization (WHO) specifies that Long COVID refers to the presence of symptoms (such as fatigue and cognitive issues) for 3 months following the initial SARS-CoV-2 infection persisting for at least 2 months after infection onset, despite the majority recovering within weeks after infection (2). It is estimated that 10%–20% of infected cases go on to develop Long COVID, impacting people across all age groups, including children, which can result in substantial difficulties for those impacted, as well as place strain on healthcare systems and society (2, 3).

Long COVID is associated with a broad spectrum of multisystem manifestations that can persist and impair functional health. Among these, cardiovascular involvement is increasingly recognized as a clinically important component of Long COVID. Accumulating evidence links SARS-CoV-2 infection to long-term detrimental effects on the circulatory system and an increased risk of persistent cardiovascular complications, including myocardial dysfunction, arrhythmias, endothelial injury, thromboembolic events, and impaired exercise tolerance (4–6). Reported sequelae include cardiovascular and cerebrovascular events (e.g., thromboembolic complications) and comorbid conditions such as type 2 diabetes mellitus and myalgic encephalomyelitis/chronic fatigue syndrome (ME/CFS). Autonomic dysfunction is also observed, including postural orthostatic tachycardia syndrome (POTS). These manifestations may persist for 12 months or longer, and longitudinal data show that some patients continue to report reduced exercise tolerance, fatigue, palpitations, and cognitive symptoms one year or even several years after SARS-CoV-2 infection (7).

Registry-based evidence indicates that the clinical presentation of persistent post-COVID symptoms varies according to hospitalization status, vaccination status, and pandemic wave, with vaccinated individuals generally showing fewer symptoms, shorter symptom duration, and lower hospitalization frequency than unvaccinated individuals (8). With the continuous evolution of SARS-CoV-2, the risk and phenotype of Long COVID have also shifted across variant-dominant periods—the Omicron era has been associated with a significantly lower prevalence of Long COVID and a different symptom spectrum compared with pre-Omicron periods (9). Current evidence suggests that COVID-19 vaccination confers partial, rather than absolute, protection against Long COVID. Vaccination has been associated with a reduced incidence of certain post-COVID symptoms, though it does not eliminate the overall risk of developing the syndrome; the magnitude of this protective effect varies according to vaccine dose, timing of administration, circulating variant, and baseline population risk (9, 10).

The persistence of symptoms likely reflects multiple, partially overlapping mechanisms. These proposed mechanisms include: (1) tissue viral reservoirs, whereby persistent viral material may provide ongoing antigenic stimulation and downstream inflammatory/coagulation signaling (11, 12); (2) immune dysregulation with or without reactivation of latent pathogens, including herpesvirus reactivation (e.g., Epstein–Barr virus) in subsets of patients (13–15); (3) microbiome/virome perturbations, in which gut–immune axis dysbiosis and barrier dysfunction may sustain inflammation and vascular dysregulation (16–18); (4) autoimmunity and molecular mimicry, potentially leading to cross-reactive autoantibodies (19–21); (5) endothelial dysfunction with microvascular coagulation, promoting hypoperfusion and tissue hypoxia (22–25); and (6) altered brainstem and/or vagal signaling, contributing to autonomic dysfunction (26, 27), among others (28). In terms of evidence strength, the most consistently reproduced clinical and biomarker associations involve immune dysregulation and autonomic dysfunction, while microbiome/virome signals are recurrent but often indirect and heterogeneous across cohorts. By contrast, tissue viral reservoirs and autoimmunity/molecular mimicry are supported in some studies, but detection rates, assay platforms, replication across independent cohorts, and causal inference remain inconsistent. Importantly, these mechanisms are not mutually exclusive and may interact dynamically; for example, viral remnants can trigger immune activation, which may in turn aggravate endothelial injury and neuro-autonomic imbalance.

Importantly, these processes are not confined to individuals with severe acute disease; they can also affect those with mild or asymptomatic infection (29). Persistent cardiovascular symptoms can markedly reduce quality of life and functional capacity and may have long-term implications for health. From a macro-level disease spectrum perspective, the vascular/cardiac dysfunction associated with Long COVID may compound the existing high baseline cardiovascular burden globally, translating into a substantial incremental burden and potential strain on healthcare resources (30). This warrants urgent attention from clinicians and researchers.

The medical community has established Long COVID's cardiovascular sequelae, but researchers need to fill important gaps regarding the exact mechanisms underlying these various conditions. The therapeutic options for Long COVID-related cardiovascular dysfunction remain limited and are supported by an evolving but still incomplete evidence base. The current management strategy focuses on treating symptoms and applying established treatments for similar non-COVID conditions (e.g., beta-blockers for tachycardia and exercise rehabilitation for deconditioning) (31). However, the distinct pathophysiology of Long COVID underscores the need for targeted research to develop interventions that address endothelial injury, immunothrombosis, dysautonomia, and persistent inflammation. Potential therapeutic approaches for Long COVID include immunomodulation therapy, antiviral strategies to clear persistent viral reservoirs, endothelial stabilization, microvascular protection, autonomic nervous system modulation, and personalized rehabilitation protocols.

The aim of this mini review was to summarize the cardiovascular burden of Long COVID, critically evaluate symptom profiles and proposed mechanisms, and synthesize current evidence on risk modifiers, including acute illness severity, vaccination status, and SARS-CoV-2 variant period, to inform clinical management and future research priorities.

## 2 Methodology

This narrative mini review was conducted by searching PubMed, Web of Science, Scopus, Embase, and Google Scholar for articles published from database inception to January 2026. The search combined terms related to Long COVID (“post-acute sequelae of SARS-CoV-2”, “Long COVID”, “PASC”) and cardiovascular outcomes (“cardiovascular”, “heart”, “vascular”, “thrombosis”, “autonomic dysfunction”, “endothelial”, “microvascular”, “myocardial”).

Inclusion criteria were: (1) studies involving human participants with persistent symptoms ≥3 months after SARS-CoV-2 infection; (2) reporting cardiovascular symptoms, objective cardiovascular findings, underlying mechanisms, risk modifiers, or management strategies in adult or pediatric populations; and (3) original research, systematic reviews, meta-analyses, registry-based analyses, consensus statements, or mechanistic studies with objective cardiovascular or biomarker endpoints.

Exclusion criteria were: (1) studies not addressing Long COVID or post-acute sequelae; (2) publications lacking cardiovascular relevance; (3) animal or *in vitro* studies without human data; (4) duplicated reports without additional insight; (5) articles not available in English; and (6) studies with insufficient methodological detail for interpretation. Case reports and preprints were considered only when peer-reviewed evidence was unavailable or when they provided unique mechanistic insight.

Two authors independently screened titles and abstracts, and full texts of potentially relevant articles were retrieved. Disagreements were resolved through discussion with a senior author. Priority was given to large cohort studies, randomized controlled trials, and high-quality systematic reviews. A total of approximately 140 articles were ultimately included in the synthesis. Data were synthesized narratively according to clinical phenotype, proposed mechanism, acute disease severity, vaccination status, variant period, and management strategy; no quantitative meta-analysis was performed because of heterogeneity in study design, outcome definitions, follow-up duration, and biomarker measurement.

## 3 Cardiovascular symptoms

Long COVID manifests with various cardiovascular symptoms affecting patients. The cardiopulmonary symptoms experienced by individuals with Long COVID include chest pain, cough, dyspnea, fatigue, palpitations, and other related symptoms (32–34). In an online survey covering 56 countries and involving 3,762 suspected and confirmed patients, Davis et al. found that within 7 months after infection, the average prevalence of heart-related symptoms was as high as 86%, which included palpitations (68%), chest pain (53%), syncope (13%), and cases meeting the diagnostic criteria for POTS (31%) (35). Similarly, Ziauddeen et al. carried out a study focusing on the symptoms of long COVID among 2,550 patients via social media platforms, and the results also showed that among patients with long COVID, the reported rate of cardiopulmonary symptoms reached 89% (36). Exhaustion, headache, breathlessness and chest pressure/tightness emerged as the most common persistent inaugural symptoms (36). Although informative, these findings from social-media-based surveys are subject to selection bias and should be interpreted alongside data from more controlled cohorts. As early as the initial stage of the COVID-19 pandemic outbreak, a research report published by Carfi et al., which focused on hospitalized patients in Italy, pointed out that the incidence of cardiopulmonary symptoms among patients with long COVID was relatively high, exceeding 43% (32). Later research, including a cohort study conducted on 1,733 hospitalized patients in China by Huang et al. revealed that six months after acute infection, 63% of the patients still experienced fatigue, 26% had dyspnea, and 5% to 9% presented with chest pain or palpitations (37). During the 12-month follow-up period, the proportions of some patients with dyspnea (30%) and chest pain (7%) increased slightly, while fatigue decreased to 20% (38). In a follow-up assessment conducted on 1,077 adult patients in the UK by Evans et al., which took place 2 to 7 months after the patients were discharged from the hospital, it was found that 48% of the patients still had fatigue, 41% had dyspnea, and 21% to 28% reported chest pain and palpitations respectively (39). These robust cohort data provide a more precise estimate of symptom burden in post-hospitalized populations. Analysis of U.S. Veterans Affairs data (>150,000 individuals) showed heightened cardiovascular risks (e.g., heart failure, arrhythmias, stroke) at one year, independent of initial COVID-19 severity, offering strong epidemiological evidence for the long-term cardiovascular sequelae of SARS-CoV-2 infection (40).

Regarding structural cardiac damage, evidence is primarily derived from imaging and autopsy studies, which, while valuable, have inherent limitations. Cardiac Magnetic Resonance Imaging (CMR) studies confirmed persistent abnormalities: cardiac involvement in 78 patients (78%) and ongoing myocardial inflammation in 60 patients (60%) (41). However, the generalizability of these findings is limited by small sample sizes and a lack of pre-infection baseline data. Autopsy studies, while subject to referral bias, provide preliminary pathophysiological insights into post-SARS-CoV-2 myocardial injury. Analysis of an early consecutive autopsy cohort comprising 80 individuals with PCR-confirmed SARS-CoV-2 infection identified suspected cardiac injury in only 5% of the cases (42). Basso et al.'s multicenter postmortem analysis of 21 specimens revealed that 14% of the specimens had myocarditis, which is typified by lymphocytic infiltration and cardiomyocyte necrosis (43). Additionally, 86% of the cases exhibited interstitial macrophage infiltration, and 19% were complicated by pericarditis accompanied by right ventricular damage (43). This discrepancy highlights the variability in pathological findings and the need for standardized histopathological definitions in the context of COVID-19.

Beyond patient-reported symptoms and structural findings, objective functional assessments have revealed measurable impairments in post-COVID-19 individuals. A controlled cross-sectional study by Nascimento et al. demonstrated that post-COVID-19 patients exhibited a 20% reduction in six-minute walk test (6MWT) distance and a 28% reduction in one-minute sit-to-stand test (1-STS) repetitions compared to uninfected controls, confirming a significant decrement in functional capacity. Crucially, this study also captured real-time cardiac hemodynamic responses during exercise using impedance cardiography. During the 1-STS, the post-COVID-19 group showed significant reductions in the stroke volume index (18%), cardiac index (21%), Contractility Index (78%), and Ejection Fraction (29%), alongside increases in Systemic Vascular Resistance (25%) and the Systemic Vascular Resistance Index (27%) (44). These findings suggest that the subjective symptoms of exercise intolerance and fatigue reported by patients may be underpinned by objective, measurable impairments in cardiac output and elevated vascular resistance during physical exertion. Notably, despite these hemodynamic alterations, muscle tissue oxygenation patterns during and after exercise were similar between groups, suggesting that peripheral oxygen delivery and consumption may be preserved even in the context of central cardiac limitations (44).

Given that Long COVID is not limited to severe/hospitalized patients, and even those with mild or asymptomatic acute infections may develop persistent cardiovascular symptoms, it is necessary to conduct stratified analyses based on the severity of acute infection. Overall, mild/asymptomatic cases more frequently present with functional or autonomic phenotypes such as palpitations, fatigue, post-exertional malaise, atypical chest pain, and autonomic dysfunction (45, 46). In contrast, severe/hospitalized patients exhibit significantly higher rates of cardiovascular symptoms and are more prone to structural or organic cardiovascular sequelae, including arrhythmias, myocardial injury, heart failure, post-thrombotic complications, and associated imaging abnormalities (34, 47). These phenotypic differences likely reflect non-exclusive contributions from autonomic dysregulation, immune activation, and microvascular/endothelial injury, with the relative weighting influenced by acute-phase injury burden, baseline cardiometabolic risk, and care-setting–dependent ascertainment. Supplementary Table S2 provides a pragmatic phenotype-oriented comparison; mechanistic assignments should be interpreted as hypotheses rather than definitive causal mappings.

Additionally, as SARS-CoV-2 variants have evolved, the phenotype and cardiovascular burden of Long COVID have also shifted. Current evidence suggests that the Delta period was associated with a higher cardiopulmonary and cardiovascular post-acute burden in several settings, whereas Alpha has often been reported as intermediate and Omicron as lower on average, although substantial symptoms (e.g., palpitations, fatigue, tachycardia) still occur, particularly among high-risk or hospitalized individuals (Supplementary Table S3) (48–51). These differences may be attributable to variations in the spike protein mutation profiles among different variants, leading to distinct cellular tropism and pathogenic mechanisms, as well as enhanced hybrid immunity levels acquired through vaccination and natural infection within the population. However, cross-variant comparisons remain strongly confounded, and few studies fully control for key factors (e.g., vaccination status, prior infection, age, comorbidities, ascertainment intensity, and follow-up duration), limiting inferences about virus-intrinsic pathogenicity. Future research requires standardized clinical endpoint definitions, matched population characteristics, and unified biomarker detection methods to more precisely quantify cardiovascular risk differences among variants.

## 4 Underlying mechanisms

**Figure 1:**
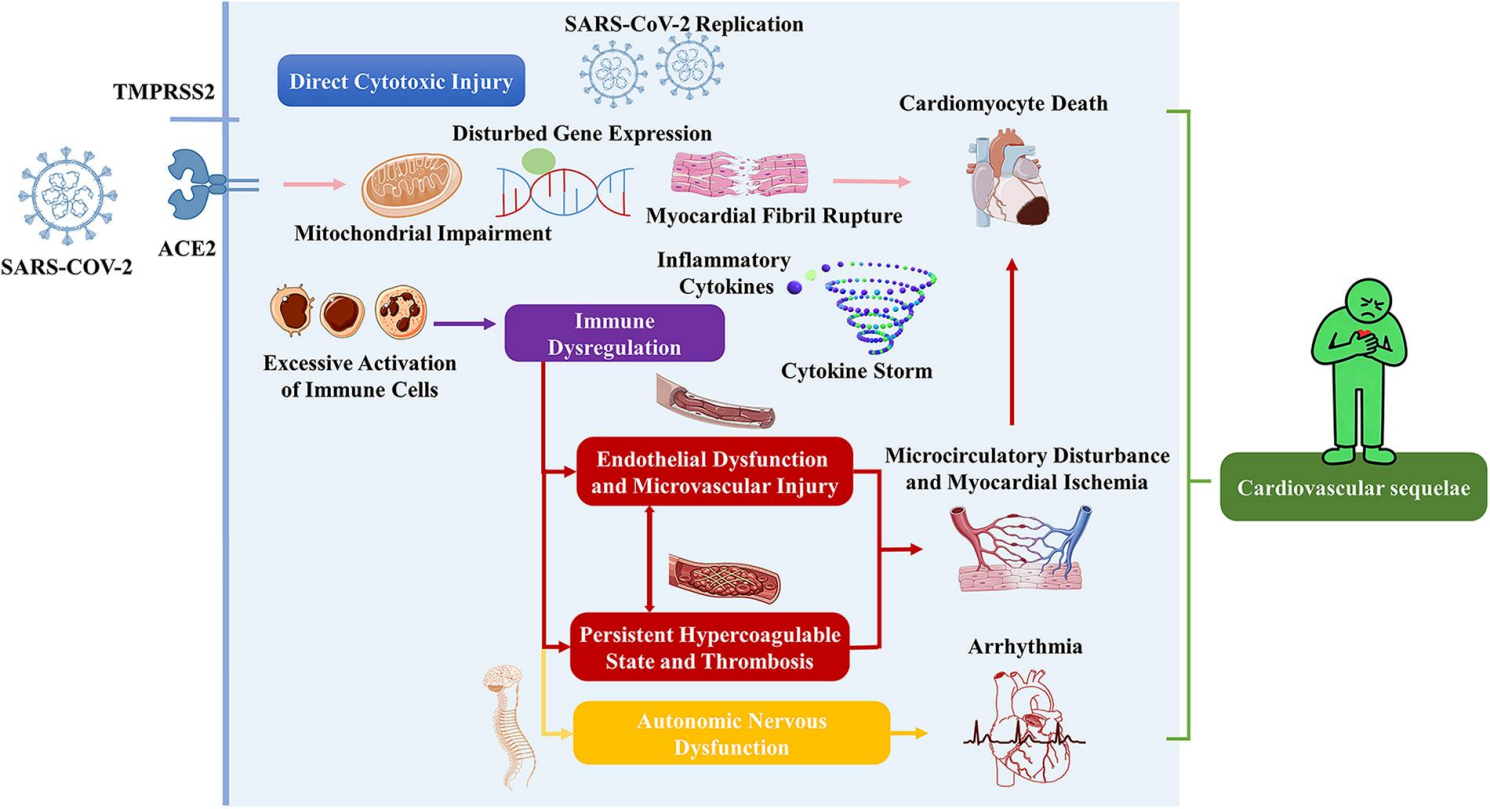
Possible core mechanisms of cardiovascular impairment in long COVID. SARS-CoV-2, Severe Acute Respiratory Syndrome Coronavirus 2; ACE2, Angiotensin-Converting Enzyme 2; TMPRSS2, Transmembrane Protease, Serine 2.

It is widely hypothesized that the cardiovascular impairment associated with long COVID arises from the synergistic interplay of multiple pathophysiological mechanisms. These include direct viral cytotoxicity, immune dysregulation culminating in cytokine release and exaggerated inflammatory responses, endothelial dysfunction coupled with microvascular injury, a persistent hypercoagulable state predisposing to thrombosis, and dysautonomia (Figure 1) (1, 52–57).

Central to this process is the role of angiotensin-converting enzyme 2 (ACE2) receptors, which facilitate SARS-CoV-2 entry into host cells (58–62). Their abundant expression in cardiac tissues renders cardiomyocytes particularly vulnerable to viral invasion. Binding of the virus to ACE2 receptors enables direct infection of myocardial cells, constituting a key mechanism of injury in COVID-19-related cardiac complications. Supporting this, a postmortem investigation of 39 individuals who had COVID-19 but no antemortem diagnosis of myocarditis revealed detectable SARS-CoV-2 genomic material in the myocardium of 61.5% of cases, with active viral replication observed in 20.9% (63). Further experimental evidence indicates that SARS-CoV-2 infection disrupts mitochondrial function, induces sarcomeric disintegration, induces cardiomyocyte apoptosis, alters transcriptional profiles, and elicits a pronounced localized immune reaction within cardiomyocytes, providing a plausible mechanistic basis for direct viral injury (64–70).

Given that ACE2 receptors are also present on neuronal cells, SARS-CoV-2 may directly impair autonomic nervous system function, contributing to dysautonomia and POTS (71–73). While the association between COVID-19 and these conditions is clinically well-recognized, the precise mechanisms—whether viral, immune-mediated, or autoimmune—remain largely hypothetical and require further mechanistic studies (74–76).

Emerging evidence strongly implicates endothelial dysfunction and microvascular injury as central, sustained drivers of Long COVID pathology (77–81). In a study involving medical centers using MRI scans of patients who had been hospitalized due to COVID-19, it was found that some patients had small vessel disease (∼9%) and myocardial infarction (∼2%) detected about 2–3 months after their infection (82). In addition to imaging findings, COVID-19 patients (including those with long COVID) often show elevated levels of cardiac troponin, which is an important indicator of myocardial injury and/or ischemia. The pathological study by Bois and colleagues strengthens the thrombogenic hypothesis—their autopsy series of 15 fatal COVID-19 cases revealed fibrin microthrombi in 80% of samples, far exceeding the incidence of acute ischemic injury (13%) or myocarditis (33%), underscoring the centrality of thrombotic pathology in COVID-19–associated cardiac damage (25). Notably, Aristotle G. Koutsiaris has placed emphasis on microvascular effects, microthrombosis, and the related tissue blood supply reduction (TBSR or SR in short) mechanism (83–85). This paradigm holds that the disruption of blood supply to peripheral tissues due to microvascular loss and hemodynamic decrease is the main cause of Long COVID symptoms (84). It also indicates that this SR mechanism is associated with seven major Long COVID symptoms, accounting for an aggregated incidence rate of 76%, which can well explain most of the reported symptoms of long COVID (85). However, it remains unclear whether these inflammatory markers are a cause or a consequence of ongoing tissue damage, and their utility as therapeutic targets or diagnostic biomarkers is still under investigation.

Persistent viral presence might be the cause of long-lasting symptoms in affected individuals. The various components of the coronavirus (SARS-CoV-2) remain in parts of the body such as heart tissues, brain cells, muscles, eyes, lymph nodes, appendix, breast tissues, liver cells, lung tissues, blood plasma, feces, and urine (28, 86). As per a research analysis conducted on Long COVID patients which included thirty-seven individuals, it was observed that the spike antigens of the virus were still detectable in about 60% of cases even after twelve months since diagnosis (11). In contrast, no trace of these antigens was found in twenty-six individuals who had fully recovered (11). This persistence is hypothesized to drive chronic immune activation. In support of this, studies have detected inflammatory cytokines—including interleukin (IL)-1, IL-16, IL-17, IL-22, interferon (IFN)-*γ*, and tumor necrosis factor (TNF)-*α*—in the blood of patients with long COVID, which may induce endothelial dysfunction, platelet activation, and neutrophil recruitment, ultimately triggering a hypercoagulable state and progressively leading to myocardial injury (28, 87–91). However, it remains unclear whether these inflammatory markers are a cause or a consequence of ongoing tissue damage, and their utility as therapeutic targets or diagnostic biomarkers is still under investigation.

The relative contributions of the aforementioned mechanisms are not fixed but are jointly influenced by factors such as host immune status, viral characteristics, acute phase severity, and the post-infection time window.

Host immune status determines the efficiency of viral antigen clearance and the capacity to maintain immune homeostasis: inadequate clearance prolongs the antigen exposure window, allowing persistent antigen stimulation and low-grade inflammation to exert greater influence in symptom persistence. While individuals prone to immune dysregulation are more susceptible to autoimmune-like responses or immune-mediated endothelial activation, which then interact with hypercoagulability, microthrombosis, and microcirculatory perfusion abnormalities. Furthermore, host genetic susceptibility may offer deeper insights into these variations. Ayyoub et al.'s review summarizes genetic evidence for persistent pulmonary vascular symptoms in long COVID, identifying specific genetic variants associated with endothelial dysfunction, coagulation/thrombosis pathways, and inflammatory responses (92). These variants may increase susceptibility to pulmonary vascular complications such as pulmonary hypertension, pulmonary thromboembolism, and pulmonary vasculitis in long COVID patients, while also influencing disease severity and progression. This perspective suggests that future efforts should prioritize large-scale, cross-population genomic validation and functional studies, integrating genetic information with clinical exposures, comorbidities, and objective endpoints to advance translational risk stratification and targeted intervention strategies.

The unique replication kinetics and immune evasion capabilities of different SARS-CoV-2 variants not only determine the intensity of acute inflammatory responses and the degree of tissue hypoxia, but may also modulate the relative weight of two core pathogenic pathways in subsequent disease progression. This occurs by influencing the duration of viral antigen exposure within the body, thereby regulating endothelial injury and coagulation dysfunction, as well as autonomic and immune dysregulation. For instance, the Delta variant is more likely to cause severe endothelial injury and hypercoagulable states, consistent with their higher hospitalization and critical illness rates (93, 94). In contrast, the Omicron variant is associated with less frequent major vascular injury but increased arrhythmias compared to negative control groups (95).

Acute severity predominantly reflects amplification of upstream injury burdens: severe or markedly hypoxic patients align more closely with pathways dominated by endothelial injury and microvascular perfusion impairment, whereas mild cases more frequently present with autonomic phenotypes such as orthostatic intolerance, palpitations, and exercise-induced symptom exacerbation (Supplementary Table S2).

Concurrently, the relative weight of different mechanisms may shift over time. A longitudinal cohort study by Huang et al. observed that symptoms like dyspnea slightly increased at one year compared to six months in some patients (38). This phenomenon cannot be explained solely by inflammatory responses and may reflect a transition from immune-inflammatory dominance to structural or functional vascular injury. Overall, available data indicate that viral antigen persistence and inflammatory signaling are detectable in at least some patients in earlier post-acute windows, whereas endothelial/microvascular abnormalities and autonomic dysfunction may persist or fluctuate over longer follow-up periods (Supplementary Table S1).

Thus, the marked heterogeneity of the cardiovascular phenotype in long COVID may fundamentally reflect differences in the relative weight of underlying mechanisms across distinct populations at different time points. This framework explains the potential therapeutic heterogeneity associated with a unified clinical pathway while underscoring the necessity for classification and risk stratification based on reproducible objective indicators. It provides direction for subsequent personalized follow-up and targeted intervention studies.

## 5 Treatment and management

**Figure 2:**
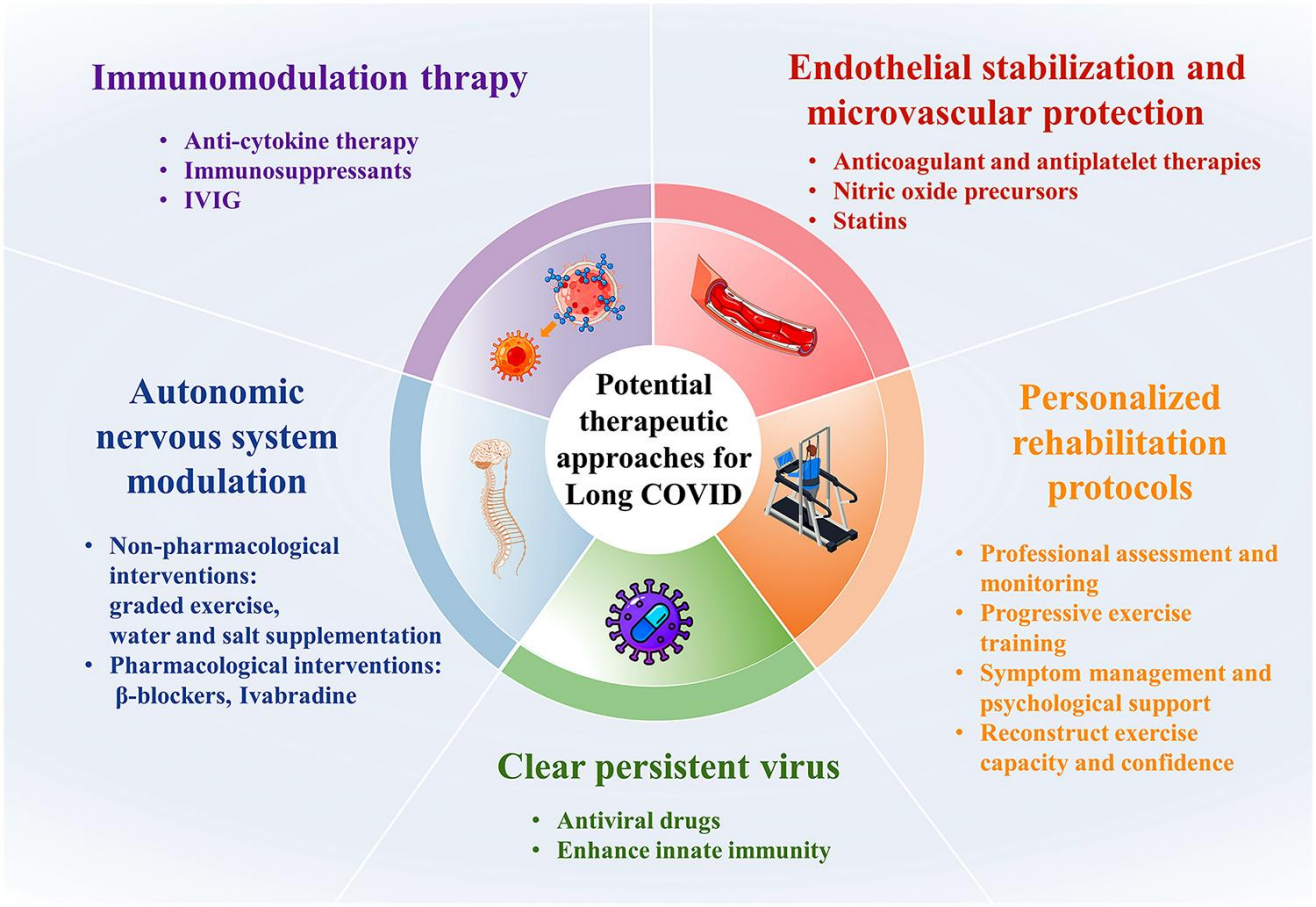
Potential therapeutic approaches for long COVID. IVIG, Intravenous Immunoglobulin.

At the present time, there exists a lack of concrete and conclusive treatment approaches for handling the physical heart-related issues stemming from Long COVID, like myocarditis/pericarditis, myocardial ischemia, as well as irregular heart rhythms known as arrhythmias; the primary approach to treatment still focuses mainly on alleviating symptoms experienced by patients (Figure 2).

### 5.1 Non-pharmacological therapy

Non-pharmacological interventions serve as the foundation of Long COVID management, particularly for symptoms like tachycardia and reduced exercise tolerance.

Structured exercise regimens have proven beneficial for symptom management after excluding heart failure and arrhythmia conditions, and physical rehabilitation leads to improved long-term outcomes (96–98). It is recommended that individualized rehabilitation programs be developed based on patients’ current activity tolerance, with avoidance of high-intensity aerobic exercise or weightlifting to prevent symptom exacerbation and post-exertional malaise (PEM) (99–101). This cautious approach is particularly relevant for athletes and physically active individuals considering a return to sport after COVID-19. Expert consensus recommendations emphasize the need for a gradual, phased return to physical activity, with careful cardiovascular evaluation prior to resuming high-intensity training, to minimize the risk of exercise-related adverse events (102, 103). These guidelines underscore the importance of balancing the well-established benefits of physical activity with the potential risks of overexertion in the post-COVID recovery phase.

Lifestyle modifications constitute another cornerstone of non-pharmacological management. Normotensive patients with normal cardiac function should appropriately increase their fluid and electrolyte intake but should prevent dehydration triggers such as alcohol, excessive heat, and overeating. Certain specific dietary patterns, such as eating small and frequent meals as well as consuming a high-fiber and high-carbohydrate diet, may help reduce postprandial blood glucose peaks and alleviate symptoms of POTS (101, 104). Additionally, a randomized crossover trial indicates that intermittent fasting and a no-sugar diet can mitigate long COVID symptoms, although these findings require replication in larger, more diverse populations (105).

### 5.2 Pharmacological therapy

Pharmacological interventions are indicated when non-pharmacological measures prove insufficient for symptom control. Drug selection should adhere to established administration standards for relevant indications, with careful consideration of potential symptom-exacerbating effects (101).

For management of tachycardia and palpitations, several options exist. Beta-blockers, non-dihydropyridine calcium channel blockers, and ivabradine are potential pharmacologic options. No head-to-head trials have demonstrated superiority of exercise training over pharmacological interventions for symptom relief in Long COVID.

Given the central role of endothelial dysfunction and thrombotic processes in Long COVID pathogenesis, antithrombotic therapy has garnered significant research interest. A hospital-based cohort study revealed that COVID-19 outpatients who were receiving outpatient anticoagulation therapy at the time of diagnosis were found to have a 43% reduced risk of hospitalization (106). Another trial showed that in patients with noncritically ill COVID-19, an initial heparin therapeutic-dose anticoagulation strategy increased the probability of survival to discharge and reduced the use of cardiovascular or respiratory support compared to usual-care thromboprophylaxis (107). The current hematologic and cardiovascular guidelines suggest that anticoagulation therapy should be prescribed to patients who have proven deep vein thrombosis (DVT) or other validated indications after hospital discharge (108–110). However, the efficacy of anticoagulation in the post-acute phase of Long COVID remains unclear and is not routinely recommended without proven thromboembolic disease. Medical research has investigated the therapeutic potential of antiplatelet therapy because platelets play an essential role in both thrombosis and inflammation. Research indicates that antiplatelet medications benefit hospitalized COVID-19 patients, yet optimal antiplatelet drug choices—together with appropriate dosages and treatment durations—need additional research for acute and Long COVID management (111–114). Nitric oxide (NO) bioavailability increases through dietary arginine supplementation, which enhances microcirculation and reduces endothelial dysfunction (115, 116). Although mechanistically sound, it is an experimental approach that requires rigorous clinical validation.

### 5.3 Interventional and advanced therapies

For patients with refractory symptoms, interventional approaches may be considered, although the evidence base remains limited.

Enhanced external counterpulsation (EECP) has emerged as a potential non-invasive intervention for Long COVID-related symptoms. Preliminary studies suggest that EECP may be beneficial in treating fatigue and cardiovascular impairment caused by Long COVID (117–119). However, these findings are derived from small, uncontrolled case series, and larger randomized controlled trials are needed to establish efficacy and define optimal patient selection criteria.

In cases where specific cardiovascular diagnoses are confirmed, standard interventional cardiology procedures apply. For example, patients with documented myocarditis, pericarditis, or arrhythmias should be managed according to established cardiology guidelines, which may include advanced imaging, electrophysiological studies, or device therapy as indicated.

## 6 Discussion

This review highlights four principal findings. First, cardiovascular Long COVID is clinically heterogeneous and may include patient-reported symptoms, functional impairment, autonomic manifestations, thromboembolic events, and objective myocardial or vascular abnormalities. Second, this heterogeneity is unlikely to be explained by a single pathway; rather, it appears to reflect overlapping contributions from viral persistence, immune dysregulation, endothelial and microvascular injury, hypercoagulability, and autonomic nervous system dysfunction. Third, clinical phenotype and symptom burden are modified by acute disease severity, baseline cardiovascular risk, vaccination status, and SARS-CoV-2 variant period. Fourth, current management remains largely symptom-based, underscoring the need for mechanism-informed risk stratification, standardized cardiovascular endpoints, and targeted interventional trials (28, 31, 40).

Evidence from mechanistic studies increasingly supports a framework of vascular and neuroautonomic regulation for long COVID-related cardiovascular complications and links clinical symptoms to abnormalities in objective measures. Using multi-b-value diffusion MRI to assess cerebral microvascular characteristics in COVID-19 survivors, van der Knaap et al. found that ICU survivors exhibited significantly greater microvascular perfusion impairment in cortical and white matter regions approximately 9 months post-infection compared to general ward patients. Notably, reduced microvascular function in white matter with normal appearance directly correlated with patients' subjective cognitive complaints. Within the ICU subgroup, acute hypoxia severity significantly associated with subsequent microvascular function metrics (120). These findings suggest acute infection severity may induce persistent cerebral microvascular dysfunction, ultimately leading to long-term cognitive impairment. They also imply microcirculatory injury as a potential common pathophysiological basis for cross-system symptoms like exercise intolerance and cognitive dysfunction. The CARTESIAN multicenter cohort provided evidence at the large-vessel level. By measuring carotid-femoral pulse wave velocity (PWV), the study found that approximately 6 months post-infection, survivors exhibited significantly higher arterial stiffness than non-infected individuals, with this difference being particularly pronounced in women and independently associated with persistent symptoms. By 12 months, PWV showed a trend toward stabilization or improvement, suggesting a potential reversible window for vascular dysfunction (121). In summary, by integrating multidimensional assessment metrics of cerebral microcirculation and large-vessel function, these studies advance the pathophysiological chain of vascular injury following COVID-19 infection—specifically, how endothelial and microvascular damage triggers persistent inflammation, leading to increased arterial stiffness and ultimately elevated long-term cardiovascular event risk—from theoretical hypothesis to quantifiable validation. This not only provides stronger evidence for understanding the elevated cardiovascular risk in Long COVID patients but also suggests that incorporating vascular function assessment into their risk stratification and follow-up management holds significant clinical value.

At the same time, acute disease severity does not fully determine the risk of cardiovascular Long COVID. Hospitalized or severely hypoxic patients may be more likely to show structural or vascular sequelae, including myocardial injury, thromboembolic complications, impaired cardiopulmonary reserve, and imaging abnormalities (34, 39, 47). In contrast, patients with mild or even asymptomatic acute infection may still develop persistent palpitations, orthostatic intolerance, post-exertional symptom exacerbation, and other autonomic or functional phenotypes (45, 46). Therefore, acute severity should be viewed as a modifier of dominant mechanisms rather than as a prerequisite for long-term cardiovascular symptoms. This interpretation is consistent with the severity-stratified phenotypes summarized in Supplementary Table S2, but the mechanistic assignments should remain hypothesis-generating because ascertainment intensity, comorbidities, and follow-up duration differ substantially across studies.

Vaccination status and variant period are also important modifiers of Long COVID risk and phenotype. Data from the Polish STOP-COVID registry indicate that headache (29.4% vs. 17.4%), arthralgia (10.3% vs. 5.4%), and dysregulation of previously controlled hypertension (18.4% vs. 11.6%) were more common among unvaccinated patients, and those vaccinated after infection had the lowest frequencies of these symptoms, suggesting that the timing of vaccination may modulate the symptom profile (10). Furthermore, Omicron-era infection reduced the risk of Long COVID by approximately 58% (OR 0.42), and acute disease severity was no longer a significant predictor, leaving female sex as the only independent risk factor (9). These findings are consistent with systematic reviews showing that vaccination reduces Long COVID risk by approximately 30%–50%, although protection is incomplete and varies by vaccine type, number of doses, and circulating variant (10). They indicate that population immunity, prior infection, vaccine dose, ascertainment bias, and follow-up duration must be incorporated into future comparative analyses.

Based on the above understanding, future research should prioritize four interconnected areas: mechanistic elucidation, diagnostic tool development, therapeutic innovation, and longitudinal risk stratification.

The primary focus is to elucidate the dynamic interactions among viral persistence, immune dysfunction, and microvascular injury, particularly in the context of varying host immune states, viral mutation characteristics, and acute phase severity. This involves deciphering the relative weight and temporal evolution of these mechanisms, especially in high-risk populations with obesity or genetic predisposition. Integrated multi-omics approaches may help delineate how viral remnants contribute to molecular mimicry, autoimmunity, and autonomic dysregulation, thereby laying the groundwork for mechanistic subtyping and target identification (122–124).

Second, there is a need to develop and validate diagnostic tools that can specifically identify cardiovascular complications associated with Long COVID. The clinical utility of vascular markers (e.g., ANG1, P-selectin), cerebral and peripheral microvascular perfusion imaging (e.g., b-value diffusion MRI, cardiac MRI), and hemodynamic responses during exercise stress (e.g., one-minute sit-to-stand test combined with impedance cardiography) will depend on standardized assays, thresholds, and reporting frameworks (44, 120, 121, 125–129). Incorporating these indicators into a core endpoint set facilitates objective symptom quantification, risk stratification, and dynamic monitoring during rehabilitation follow-up.

Third, therapeutic innovation should prioritize developing precision intervention strategies targeting distinct mechanism-dominated subgroups, overcoming the current limitations of primarily symptomatic treatment. Given the significant heterogeneity of phenotypes, clinical studies should employ stratified randomized controlled designs based on reproducible objective metrics to identify optimal intervention combinations under different mechanism modules, thereby avoiding efficacy disparities resulting from a one-size-fits-all approach. Currently, research and trials for antiviral drugs and monoclonal antibodies targeting long COVID are underway (130, 131). However, progress is constrained by incomplete mechanistic clarity and the syndrome's multisystem heterogeneity. In the interim, management may rely on repurposed therapies (e.g., statins for vasoprotection and *β*-blockers for autonomic symptoms) while mechanism-based interventions are developed.

The integration of artificial intelligence (AI) into Long COVID research represents a promising but still nascent frontier. AI-assisted prediction models integrating electronic health records (EHR) data with multi-omic biomarker profiles may support early risk stratification and personalized intervention planning. Machine learning algorithms could potentially identify unrecognized disease subtypes, predict treatment response, and optimize clinical trial enrollment. However, it is important to acknowledge that the application of AI in Long COVID therapeutics remains largely theoretical at present. Robust, prospectively collected datasets are prerequisites for training and validating clinically useful AI tools, and these are only beginning to emerge. Furthermore, model interpretability, algorithmic bias, and data privacy concerns must be carefully addressed before AI can be safely integrated into clinical decision-making.

Ultimately, longitudinal studies should quantify long-term cardiovascular risk trajectories after SARS-CoV-2 infection, especially in individuals with pre-existing cardiovascular disease. Large-scale health-record analyses (e.g., US Veterans Affairs databases) suggest elevated risks of heart failure and arrhythmias among patients with pre-existing conditions (34). Extending these observations to five- and ten-year horizons will be essential for understanding the true chronic disease burden imposed by the pandemic.

Advancing the field will require a coordinated framework integrating viral surveillance, adaptive clinical trials, and interoperable data platforms. Such infrastructure can accelerate the transition from symptom-based care toward mechanism-informed precision strategies for Long COVID. In conclusion, there is an urgent need to develop targeted, mechanism-informed, and personalized strategies for Long COVID–related cardiovascular complications to improve long-term outcomes.

### 6.1 Strengths of the study

This review has several strengths. First, it integrates clinical symptom profiles, objective cardiovascular assessments, mechanistic evidence, and management strategies into a unified framework on cardiovascular Long COVID. Second, it explicitly considers the roles of acute disease severity, vaccination status, and SARS-CoV-2 variant period as clinically relevant modifiers. Third, the structure of the review, including the use of phenotype-oriented supplementary tables, helps to clarify a complex and heterogeneous body of literature for clinical readers.

### 6.2 Limitations of the study

This review has several limitations. First, it is not based on a systematic review but rather a narrative mini review, which may lead to bias in the selection of research due to publication availability and author judgment. Second, the long-term COVID-19-related databases use a wide range of methodologies such as case definition, follow-up period, vaccination status, the timing of variant viruses, pre-existing diseases, and cardiovascular results assessment. Third, many of the proposed mechanisms here have not yet been proven to have a causal relationship, and most of the potential biomarkers and imaging test results need to be verified prospectively.

## 7 Conclusion

This mini review shows that cardiovascular Long COVID represents a clinically important and heterogeneous burden characterized by symptoms such as chest pain, palpitations, fatigue, exercise intolerance, dysautonomia, thromboembolic complications, and, in some patients, objective myocardial or vascular abnormalities. The available evidence supports a multi-mechanism framework in which viral persistence, immune dysregulation, endothelial dysfunction, microvascular injury, hypercoagulability, and autonomic nervous system dysfunction interact dynamically rather than acting as isolated pathways. The relative contribution of each mechanism is influenced by host immune status, viral characteristics, acute-phase severity, vaccination status, baseline cardiovascular risk, and the post-infection time window, leading to marked phenotypic heterogeneity.

Based on these findings, the following clinical recommendations are proposed: (1) cardiovascular evaluation should be considered in patients with persistent post-COVID-19 symptoms, including autonomic function testing and imaging where indicated; (2) management should be phenotype-driven and multidisciplinary, incorporating tailored exercise rehabilitation, pharmacological control of heart rate and symptoms, and anticoagulation only when thromboembolic disease is confirmed; (3) vaccination should be encouraged as a preventive measure to reduce Long COVID risk; and (4) future research should prioritize mechanism-based subtyping and randomized trials of targeted interventions.
