# Supplementary material for: Unraveling the cardiovascular burden of long COVID: symptom profiles, underlying mechanisms, and clinical management insights
Source: Front Cardiovasc Med. 2026 Jun 1;13:1786633. doi: 10.3389/fcvm.2026.1786633 (PMC13276799; doi:10.3389/fcvm.2026.1786633)
Supplement: Supplementary file 1 [file Datasheet1.docx]

**Supplementary Material**

This file includes:

Supplementary Tables

**Supplementary Tables**

**Supplementary Table 1. Core Pathological Mechanism Hypothesis of Cardiovascular Symptoms in Long COVID**

| Underlying mechanism | Core hypothesis | Key evidence/ representative literature | Evidence strength^a^ (as of 2025) | Time dynamics and clinical correlations^b^ |
| --- | --- | --- | --- | --- |
| Tissue viral reservoirs | Persistence of viral reservoirs in tissues may sustain antigenic stimulation, potentially promoting inflammatory and coagulation pathways. | Viral proteins/antigens detected in plasma and in selected tissue-biopsy studies among Long COVID patients (6, 7). | Moderate | Signals are reported in some cohorts in earlier post-acute windows; links to systemic inflammation are inconsistent across studies and may depend on sampling site and assay sensitivity. |
| Immune dysregulation | Persistent activation, exhaustion, or imbalance of innate/adaptive immunity may contribute to cardiovascular symptoms and may co-occur with herpesvirus reactivation (e.g., EBV). | Immune activation/ exhaustion signatures; possible concomitant herpesvirus (e.g., EBV) reactivation (8-10). | Moderate | Observed across the post-acute course; subgroup patterns (e.g., sex differences, baseline immune status) are suggested but not uniform, and are sensitive to cohort composition and outcome definitions. |
| Microbiome/ virome perturbations | Dysbiosis of the gut-immune axis, compromised barrier integrity, and altered metabolite profiles may contribute to chronic inflammation and vascular dysregulation. | Altered gut microbiota composition correlates with systemic inflammatory markers (11). | Low-Moderate | Gut microbiome signals may evolve over time; associations with cardiovascular symptoms are indirect in many studies and may reflect systemic inflammation or comorbidity rather than a specific cardiac pathway |
| Autoimmunity and molecular mimicry | Structural homology between viral antigens and host proteins may promote cross-reactive autoantibody formation and downstream vascular effects. | Bioinformatic and serologic studies suggest molecular mimicry (14, 15); cohort studies report increased post-infectious autoimmunity risk (16). | Low-Moderate | Autoantibodies and autoimmune signals may be more apparent in some persistent cases; temporal ordering and causality remain uncertain, with heterogeneity by assay platform and case definition. |
| Endothelial dysfunction and microvascular coagulation | Endothelial injury and hypercoagulability may promote microthrombosis, contributing to hypoperfusion and tissue hypoxia. | Histological evidence of microthrombi; elevated endothelial injury markers and coagulation abnormalities (17, 18, 20). | Moderate-High | Microvascular abnormalities are reported from early to later phases; persistence and clinical correlation are variable, and may be enriched in individuals with severe acute illness or pre-existing vascular risk. |
| Brainstem and/or vagal nerve dysfunction | Brainstem injury/inflammation and/or vagus-nerve dysfunction may precipitate autonomic dysregulation relevant to palpitations, orthostatic intolerance, and exercise intolerance. | High prevalence of POTS and autonomic disorders; neuroimaging and neuropathological studies have described multi-region abnormalities (21, 22) | Moderate | Autonomic symptoms can fluctuate and may persist; links to structural brainstem lesions are not consistently demonstrated, and clinical patterns may differ by age and baseline autonomic vulnerability. |

^a^"Evidence strength" was evaluated using a simplified GRADE framework (High / Moderate / Low). High: consistent findings across multiple well-controlled cohorts and/or systematic reviews/meta-analyses with clinically relevant outcomes. Moderate: repeated associations exist but are limited by heterogeneity, residual confounding, indirectness, or measurement variation. Low: small samples, major design limitations, inconsistent findings, or primarily mechanistic/associational evidence without clear clinical linkage.

^b^"Time dynamics" statements are trend-level summaries across heterogeneous cohorts and should not be interpreted as uniform trajectories.

Abbreviations: EBV, Epstein–Barr virus; GRADE, Grading of Recommendations Assessment, Development and Evaluation; POTS, postural orthostatic tachycardia syndrome.

**Supplementary Table 2. Comparative Analysis of Cardiovascular Long COVID Phenotypes Stratified by Acute Illness Severity**

| Acute disease severity | Predominant cardiovascular clinical phenotypes | Proposed dominant mechanism |
| --- | --- | --- |
| Mild/asymptomatic infection | Functional/autonomic: fatigue, palpitations, chest pain, orthostatic intolerance, post‑exertional malaise | - Brainstem and/or vagal nerve dysfunction - Immune dysregulation |
| Severe/hospitalized infection | Structural/organic: myocarditis, infarction, heart failure, thromboembolism, arrhythmia | - Microbiome/virome perturbations - Immune dysregulation |

**Supplementary Table 3. Comparison of Cardiovascular Long COVID Features Across Different SARS-CoV-2 Variants**

| SARS-CoV-2 Variant | Related cardiovascular burden | Evidence certainty and key confounders |
| --- | --- | --- |
| Ancestral (progenitor) strain | Moderate to high (baseline estimates vary by setting and case definition) | Moderate: limited by heterogeneous diagnostic criteria and early-pandemic testing constraints; treatment protocols evolved rapidly. |
| Alpha | Moderate (often reported lower than Delta, but estimates vary) | Low-Moderate: limited by sample size, variant misclassification, and inconsistent follow-up. |
| Delta | Moderate to high (often reported higher cardiopulmonary complications than Alpha/Omicron in several settings) | Moderate: strongly confounded by vaccination coverage, reinfection dynamics, healthcare strain, and changing hospitalization thresholds. |
| Omicron | Lower on average (but not negligible; high incidence may yield substantial absolute case counts) | Moderate: influenced by pre-existing population immunity, repeated infections, ascertainment bias, and shorter follow-up windows. |

Abbreviations: COVID-19, coronavirus disease 2019; SARS-CoV-2, severe acute respiratory syndrome coronavirus 2.
